# A survey of graduates’ perceptions on a Chinese medical school’s traditional and reform curricula

**DOI:** 10.1007/s40037-016-0282-4

**Published:** 2016-07-19

**Authors:** Renslow Sherer, Hongmei Dong, Feng Yu, Jingyi Fan, Jinxin Li, Ivy Jiang, Brian Cooper, Jonathan Lio, Yunfeng Zhou, Jiong Yang, Baoping Yu, Xiangting Yu

**Affiliations:** 1 Section of Infectious Diseases and Global Health, Department of Medicine, University of Chicago, Chicago, USA; 2 School of Medicine, Wuhan University, Wuhan, China; 3 Zhongnan Hospital, Wuhan University, Wuhan, China

**Keywords:** Medical education reform, Curricular change, China, Graduation survey, International medical education collaborations

## Abstract

A medical school in China engaged in reform in 2009 by adapting the medical curriculum of the University of Chicago, USA. Freshmen volunteered for the reform and 50 were randomly selected while the rest remained in the traditional curriculum. In 2014 a study was conducted to determine whether the views of traditional and reform curriculum students on their respective educational experience differed and to identify reform areas that needed improvement.

A survey was administered to graduating students to gauge their views on basic science and clinical science education, clerkships, general medical education, and readiness for residency training. Frequency distributions, Mann-Whitney U tests, and Chi-squared tests were used for analysis.

Reform curriculum students were more positive about their basic science and clinical science instruction. Clerkships were only somewhat satisfactory to students in both curricula. Reform curriculum students were more likely than those following the traditional curriculum to consider instruction in clinical decision-making and patient care as ‘adequate’. Instruction in population health was considered inadequate by the majority of students. Reform curriculum students were more confident about their preparedness for residency.

The traditional curriculum was in need of reform. Reform has been effectively implemented and has increased student confidence and satisfaction with their education although there is room for improvement.

## Introduction

For over a decade, Chinese and Western scholars have been calling for China’s medical education to reform by restructuring curricula and adopting newer pedagogical methods. They have pointed out weaknesses in China’s traditional approaches, including a heavy reliance on didactic lectures, a role of learners as passive recipients of information, courses that are department and discipline based, a lack of clearly stated educational objectives, poorly developed assessment systems, inadequate clinical experience, and a lack of teacher training [1–5]. Recommendations have been proposed to address these weaknesses, often after comparing the medical curricula in China and North America or Western Europe. For example, based on a comparative analysis of curricular components in the US and China, Sun and Zhao [5] argued that Chinese medical colleges should revise curricular frameworks by adopting an organ/system-based approach, integrating courses where appropriate, devoting more attention to some subjects such as community medicine and mental health, adding early and more clinical practice, and fostering students’ active learning via problem-based methods and independent or group work.

Medical curricular changes have swept across China, and key trends in the reform are in line with the recommendations mentioned above [4]. In addition, input from expertise outside the country is common practice [4, 6]. These changes are also in keeping with global trends in medical education reform. A review of the literature on medical education in emerging market economies (including such countries as Brazil, China, Mexico, and Russia) suggests that models of medical curriculum planning in many countries now display similar characteristics [7].

It was in this context of national and global reform that the School of Medicine at Wuhan University, China, launched its curricular innovations with the assistance of the School of Medicine of the University of Chicago. Wuhan University medical school had approximately 1,300 undergraduate students in a five-year programme, a basic science faculty of various disciplines, and a clinical faculty who work in two teaching hospitals. The teaching approaches before the reform had been traditional, characterized by discipline-based and department-controlled courses and large lectures. To prepare for the reform, the faculty of Wuhan University and faculty consultants of the University of Chicago assessed the existing curriculum and pedagogy by using expert panels, and student and faculty surveys. The general conclusion was that the traditional curriculum and pedagogies at Wuhan University displayed all the shortcomings as previously described. Informed by Wuhan University’s assessment of its needs and by a literature review of current medical education approaches, the leadership of Wuhan University decided to adopt the University of Chicago’s medical curriculum and pedagogy with modifications to suit its own context. The University of Chicago’s medical curriculum includes the following key elements:

- Integrated basic science courses in years 1 and 2, with each course using clinical cases or vignettes to highlight clinical application,
- The integrated *Clinical Pathophysiology and Treatments *course in year 2 to introduce students to clinical medicine and to help develop students’ clinical reasoning,
- Clerkships in years 3 and 4,
- Communication, clinical skills, and patient contact training in all years,
- Emphasis on group and independent learning,
- A strong emphasis on formative assessment.

Leadership at Wuhan University medical school identified reform course directors and convened them into a reform committee. Protected time for reform activities was provided for the course directors, who led iterative curriculum review and redesign initiatives. Course directors and faculty members were introduced to the University of Chicago medical curriculum and pedagogies through training workshops or visits to Chicago.

In 2009, Wuhan University implemented a pilot reform curriculum alongside its traditional curriculum. Each year since then, freshmen volunteered to be in the reform curriculum, and 50 were randomly selected to join the reform. The rest of the freshmen (about 250) remained in the traditional curriculum. Wuhan University’s undergraduate medical education is a five-year programme, with the first three years focusing on preclinical coursework and the final two years on clinical clerkships.

Wuhan University’s curriculum reform had the following main characteristics. First, lecture time in basic and clinical sciences was reduced by about 40 %, with a corresponding increase in small-group and independent learning. Second, basic science courses were integrated where appropriate and their relevance to clinical medicine was made apparent via clinical vignettes to illuminate mechanisms of disease pathogenesis. Third, the University of Chicago’s clinical medicine course, *Clinical Pathophysiology and Therapeutics*, which introduces students to the clinical pathophysiology and therapeutic modalities of selected diseases linked to ten physiological systems, was adapted and first implemented at Wuhan University in 2011 with year 3 students. The *Clinical Pathophysiology and Therapeutics* course achieves the integration of basic sciences as pathogenesis into the clinical sciences with an organ system approach taught jointly by pathologists and clinicians. Fourth, several new courses were created for reform curriculum students including research and doctor-patient communication, and coursework was increased for community medicine and medical ethics. Fifth, formative assessments in the form of periodic quizzes and group work evaluations were added to the traditional summative final exams to inform teaching and learning. Both formative and summative assessments in the reform curriculum reflected the new curricular goals of integration, clinical relevance, collaborative learning, and the development of thinking. Finally, by the time the first reform class entered clinical rotations (in years 4 and 5), the clerkships had been restructured, and reform and traditional curriculum students rotated together. In other words, the two groups of students were exposed to similar experiences in clinical rotations. The new clerkships were more systematic in structure using more uniform guidelines across departments. As a supplement to clinical lectures and as a way to help students think like physicians, a new course called *Clinical Thinking* was created and conducted via teacher-led discussions using real cases. The main difference between the clerkship training in the reform and the traditional curriculum was that the community medicine clerkship was mandatory for the former group while elective for the latter. For a description of the new curriculum, pedagogy, and assessment practice, please also see a survey study by the Wuhan University medical faculty [8].

Two previous surveys, one with students and the other with faculty, were conducted to gauge participants’ views of the reform during the third year of its implementation. In one study, year 3 students were asked to evaluate their preclinical curricula [9] and in the other, faculty members who taught both traditional and reform curricula evaluated the curricula’s preclinical portions [8]. Both of those studies found overwhelming support for the reform on the part of its participants. A summary of major lessons learned from Wuhan University’s curricular and pedagogical overhaul was recently published [10].

A relatively recent development in medical education is the application of the principles of evidence-based medicine in the form of ‘best-evidence medical education’; that is, the implementation of approaches to education based on the best evidence available [11, 12]. Evidence comes from two main sources – studies of learning outcomes and studies of students’ and graduates’ perceptions on the effectiveness of training programmes [11]. Perceptions are as important a measure of effectiveness as learning outcomes. In North America and some European countries, medical faculties are required to conduct periodic self-evaluation, including opinion surveys, for accreditation purposes [11]. Eyal and colleagues also maintained that such evaluations should include the perceptions of students, graduates, and faculty [11]. Regarding the implementation of a new programme, two questions should be answered: Was this programme implemented as planned, and how can it be improved [13]?

This study aimed at assessing the results of the reform by examining graduates’ perceptions of their entire five-year education. Specifically, we conducted a survey on the views of graduates trained in the reform and the traditional curricula in order to answer the following questions at the end of the new curriculum’s first full cycle of implementation. First, what were students’ assessments of the reform and traditional curricula? Second, from the reform curriculum students’ response, were the intended new elements present in their curriculum? Third, were the two groups of students’ evaluations of their respective educational experience significantly different in terms of basic science, clinical science, general medical education, preparedness for residency, and overall satisfaction with their medical school education? Finally, what areas of the reform curriculum need improvement?

## Methods

### Participants

As of the year of this study (2014), about 300 students graduated each June from the five-year programme at Wuhan University. Of the 2014 graduates, 50 had been trained in the reform curriculum since 2009 while the rest were educated under the traditional paradigm. For the first three years of medical school, when basic science classes and introduction to clinical medicine courses were offered, reform and traditional curriculum students were educated under two different paradigms. By the time the reform curriculum students entered their clinical rotation years, all clerkships had been restructured, with newly added features such as lectures on clinical thinking, formative assessments of students’ performance, and a new rotation schedule. As a result of the innovations, reform and traditional curriculum students rotated together, taught by the same faculty following the same teaching guidelines. At the time of this study, the first class of reform curriculum students had just fulfilled all their programme requirements and were about to graduate along with their traditional curriculum peers. All graduating students were informed of the survey and were invited to participate. The actual number of student respondents is given in the Results section.

### The survey instrument, data collection, and data analyses

A survey was developed based on the Graduation Questionnaire of the Association of American Medical Colleges (AAMC, 2013) [14]. Many of the AAMC Graduation Questionnaire items on curriculum and instruction were included in the survey (©2014 AAMC; used by permission). The questionnaire focuses on critical issues for medical students and educators and is widely used in the US and Canada for medical schools to capture information to help guide curricular reform and programme improvement. We also added questions that were important and relevant to unique features of the medical curricula of Wuhan University. Most of the survey items were five-point Likert-scale questions measuring satisfactions, where 1 stands for ‘strongly disagree’, 2, ‘somewhat disagree‘, 3, ‘neither agree nor disagree’, 4, ‘somewhat agree’, and 5, ‘strongly agree’. Some item sets asked students to rate courses on a four-point scale where 1 stands for ‘poor’, 2, ‘fair’, 3, ‘good’, and 4, ‘excellent’. There are also items on demographics and career choices, as well as open-ended questions that asked students to write comments. The survey was in Chinese only.

The survey asked students to assess their medical education focusing on four aspects: the preclinical years, including instruction and learning in the basic sciences and clinical sciences; the training in clinical settings in years 4 and 5; satisfaction with general medical education; and the degree to which students felt ready for residency training.

All students were notified of the survey via email and were asked to participate. The email notification made it clear that the survey was voluntary and anonymous. This was the first time a graduation survey had been held among graduating students at this medical school and the school administration considered it appropriate to make it optional for students. Those who volunteered to participate answered the survey on paper in two classrooms.

Analyses consisted of frequency distribution for each variable when applicable. To arrive at percentages of students who indicated satisfaction with a given topic, ratings of ‘somewhat agree’ and ‘strongly agree’ were combined. When appropriate, the attitudes of students in the two curricula were compared using Mann-Whitney U tests or Chi-squared tests. Because some variables were ordinal data (those measured with the Likert scale) and because tests for normality of distribution revealed that this assumption for parametric tests was violated in many cases, we considered the Mann-Whitney U test, which is nonparametric and does not assume normal distribution, to be appropriate in order to yield more accurate results. For Chi-squared tests, ratings of ‘poor’ and ‘fair’ were combined, as were ratings of ‘good’ and ‘excellent’. We applied Bonferroni corrections to multiple comparisons in order to set rigorous significance levels. Effect sizes (Cohen’s *d*) are also provided for significant and nonsignificant differences. More detailed information on survey items and analysis methods is given in each section in the Results below.

This research was approved by the University of Chicago Biological Science Division Institutional Review Board and by Wuhan University Health Science Center Ethics Committee.

## Results

A total of 178 students participated in the survey, with 145 from the traditional and 33 from reform curriculum. The total response rate was 59.3 %. When we used 0.05 as the significance level for statistical tests, some of the differences were statistically significant but small and may be practically insignificant. We therefore applied Bonferroni corrections to adjust for type I error.

## Students’ overall evaluation of basic science learning

Analyses show that students following the reform curriculum were generally more positive about their basic science courses than traditional curriculum students were with theirs, with 70 % or more reform curriculum students agreeing with each of the given statements. Reform curriculum students’ mean scores were also higher, and four of the differences were significant (the significance level was *p* < 0.01 based on Bonferroni corrections). Percentages of students who indicated ‘somewhat agree’ or ‘strongly agree’, mean scores, standard deviations, hypothesis test (Mann-Whitney U) results, and effect sizes are presented in Table 1. The statistically significant differences (items 1–4) had medium to large effect sizes, and the effect size for item 5, though relatively small, was not trivial. These results suggested that reform curriculum students were more likely to be satisfied with their basic science learning.

**Table 1:** Evaluation of basic science education

Items		1) Basic science content objectives were made clear to students	2) Basic science content was sufficiently integrated across basic science courses	3) Basic science content objectives and examination content matched closely	4) Basic science content had sufficient illustrations of clinical relevance	5) Basic science content provided relevant preparation for clerkships
% of agree	TC	57.9	37.9	56.6	43.4	65.5
	RC	78.8	72.7	81.8	69.7	72.7
TC	Mean	3.48	3.20	3.46	3.26	3.64
	SD	0.973	0.925	0.936	0.993	0.926
RC	Mean	4.03	3.91	4.03	3.85	3.91
	SD	0.770	0.843	0.728	0.906	0.843
U (*p*)		*1,657.5 (0.003)	*1,401.5 (<0.0001)	*1,587.5 (0.001)	*1,616.5 (0.002)	2,041.0 (0.153)
Cohen’s *d*		−0.627	−0.802	−0.680	−0.621	−0.305

*stands for significant difference

## Evaluation of the clinical science subjects

Students used a four-point scale to rate the quality of instruction in their respective clinical science subjects (20 in total). Chi-squared tests found no significant difference in the views of the two groups of students with a corrected significance level of *p* < 0.0025 (for this test, the ratings of ‘poor’ and ‘fair’ were combined, as were ratings of ‘good’ and ‘excellent’). However, the percentages of students who selected ‘good’ or ‘excellent’ indicated that reform curriculum students (73 % to 100 %) appeared to be more satisfied than their traditional curriculum peers (61 % to 88 %). This was especially the case for the following subjects (reform versus traditional curriculum students): internal medicine (100 % vs. 83.2 %), surgery (97 % vs.83.4 %), obstetrics/gynaecology (97 % vs. 81.9 %), emergency medicine (93.9 % vs. 72.4 %), infectious diseases (90.9 vs. 71.7 %), neurology (90.9 % vs. 73.6 %), imaging (96.9 % vs. 83.3 %), and dermatology (87.9 % vs.64.6 %).

Although the *p*-values were not statistically significant for the eight subjects above, their effect sizes were between small and medium, indicating that these results may be practically significant. Specifically, χ^2^, *p*, and Cohen’s *d* for these subjects were: internal medicine (χ^2^ = 6.413; *p* = 0.011; *d* = 0.387), surgery (χ^2^ = 4.071; *p* = 0.044; *d* = 0.306), obstetrics/gynaecology (χ^2^ = 4.689; *p* = 0.030; *d* = 0.329), emergency medicine (χ^2^ = 6.909; *p* = 0.009; *d* = 0.402), infectious diseases (χ^2^ = 5.317; *p* = 0.021; *d* = 0.351), neurology (χ^2^ = 4.514; *p* = 0.034; *d* = 0.323), imaging (χ^2^ = 3.940; *p* = 0.047; *d* = 0.301), and dermatology (χ^2^ = 6.803; *p* = 0.009; *d* = 0.399).

Only reform curriculum students were offered the introductory clinical science course *Clinical Pathophysiology and Therapeutics*. These students’ evaluation of this course showed that it was a great success: 31 of the 33 students (93.9 %) considered it ‘excellent’ and one student thought it was ‘good’ (3 %). The other student did not answer this question.

## The quality of clinical clerkships

Using a five-point scale, students rated clerkships on seven dimensions of teaching. The results of six specialties (internal medicine, surgery, obstetrics/gynaecology, paediatrics, family medicine, and psychiatry) are presented in Table 2. Overall, mean scores suggest that the clerkships were considered only somewhat satisfactory by students, and the percentages of students who ‘agree’ showed mixed results. Community and psychiatry clerkships received relatively low ratings across all dimensions with ‘agree’ rates of 66 % or lower, and dimension one (clarity of objectives) and dimension two (assessments of performance) received satisfaction rates of 66.1 % or lower for all the six specialties.

**Table 2:** Evaluation of clerkships

Items	1) I received clear learning objectives for the clerkship	2) My performance was assessed against the learning objectives	3) I had an opportunity to follow a variety of patients (with different conditions)	4) A faculty member observed me taking patient history	5) A faculty member observed me performing physical examinations	6) Faculty members provided me with sufficient feedback	7) Faculty members provided effective teaching
Internal medicine	3.53 (1.121) 54.6 %	3.53 (1.074) 54.6 %	3.82 (0.941) 70.3 %	3.77 (1.057) 67.1 %	3.76 (1.025) 65.9 %	3.64 (1.018) 62.9 %	3.87 (1.026) 70.3 %
Surgery	3.65 (1.069) 60.1 %	3.70 (1.001) 62.9 %	3.87 (0.885) 68.5 %	3.77 (0.985) 65.7 %	3.80 (1.016) 66.8 %	3.83 (0.938) 68.6 %	3.94 (0.931) 72.4 %
Obstetrics, gynecology	3.73 (1.025) 60.7 %	3.79 (0.966) 66.1 %	3.89 (0.899) 67.9 %	3.82 (0.975) 63.1 %	3.86 (0.947) 68.5 %	3.78 (0.950) 65.5 %	3.93 (0.932) 72.1 %
Pediatrics	3.81 (0.992) 64.6 %	3.81 (0.975) 64.6 %	3.87 (0.933) 68.2 %	3.91 (0.937) 70.6 %	3.86 (0.933) 67.6 %	3.91 (0.949) 72.4 %	3.97 (0.915) 72.4 %
Community medicine	3.55 (1.172) 65.5 %	3.57 (1.126) 60.4 %	3.47 (1.112) 53.4 %	3.52 (1.128) 56.9 %	3.51 (1.120) 57.9 %	3.47 (1.127) 53.4 %	3.48 (1.157) 56.6 %
Psychiatry	3.63 (0.966) 60.3 %	3.56 (0.944) 57.8 %	3.51 (0.958) 53.7 %	3.55 (1.010) 54.4 %	3.48 (0.981) 50 %	3.71 (0.958) 64.7 %	3.65 (0.947) 61.1 %

Mean, SD (in parentheses), and percentages (%) of students who ‘agree’ with given statements

## Evaluation of general medical education

As evaluation of general medical education, students rated the amount of instruction that was devoted to key areas of clinical competencies and population health by indicating whether instruction in 26 given areas was inadequate, appropriate, or excessive. Fourteen of the items were related to clinical decision-making and patient care, such as patient interviewing skills, physical examination, diagnosis, clinical reasoning, disease management, care of ambulatory patients, health education, and communication skills. The remaining items were on population health, such as public health, community medicine, disease prevention, epidemiology, health policy, health surveillance, and global health.

Percentages of students who chose ‘inappropriate’, ‘appropriate’, and ‘excessive’ were calculated, and Chi-squared tests were conducted to identify associations between students’ curriculum types and their likeliness to choose ‘appropriate’. Results showed that some areas of clinical competencies, namely patient interviewing skills, examination skills, and disease diagnosis, were rather satisfactory to both reform and traditional curriculum students, for more than 75 % of students from each group considered the amount of instruction in these areas appropriate. Students’ satisfaction appeared to be particularly low with respect to care of ambulatory patients, health education, and care of geriatric patients, as only 63 % or less students considered the amount of instruction in them appropriate. Another finding is that greater proportions of reform than traditional curriculum students chose ‘appropriate’ for 11 of the 14 areas related to clinical decision-making and patient care, especially diagnosis and clinical reasoning. For disease diagnosis, 75.2 % of traditional curriculum students and 93.9 % of reform curriculum students chose ‘appropriate’ (insignificant difference, χ^2^ = 5.64, *p* = 0.018, *d* = 0.362), while for clinical reasoning, 59.3 % of traditional and 93.9 % of reform curriculum students chose ‘appropriate’ (significant difference, χ^2^ = 14.31, *p* < 0.0001, *d* = 0.591). No other significant differences were found between the reform and traditional curriculum regarding clinical abilities and patient care (the significance level was set at 0.0019 after Bonferroni corrections).

As for topics in population health, large percentages of students deemed instruction in these areas ‘inadequate’, especially global health (about 61 % for both groups of students) and health surveillance (62 % of traditional and 55 % of reform curriculum students). Only one significant difference was found between the two groups: community medicine, which was deemed adequate by 37.9 % of traditional curriculum students and 72.7 % of reform curriculum students (χ^2^ = 13.19, *p* < 0.0001, *d* = 0.566).

## Students’ preparedness for residency and overall satisfaction with medical education

On a five-point scale, students assessed their readiness for entering residency training (items 1–7 in Table 3) as well as their satisfaction with their overall medical school education (item 8). The mean scores of students following the reformed curriculum were all higher than those following the traditional curriculum, and reform-traditional differences in two of the seven items about residency training were significant based on the corrected significance level of *p* < 0.0071. Effect sizes for items 1, 5, 6, and 7 were close to medium, suggesting the possibility of substantive differences though a statistically significant difference was absent.

Reform curriculum students were also significantly more satisfied with their medical school education overall. The percentages of participants who chose ‘agree’ with each statement also indicated a higher level of satisfaction on the part of the reform curriculum students.

**Table 3:** Students’ views on their readiness for residency training (items 1–7) and overall satisfaction with education (8)

Items		1) I am confident that I have acquired the clinical skills required to begin a residency programme	2) I have the fundamental understanding of common conditions and their management encountered in the major clinical disciplines	3) I have the communication skills necessary to interact with patients and health professionals	4) I have basic skills in clinical decision making and the application of evidence based information to medical practice	5) I have a fundamental understanding of the issues in social sciences of medicine	6) I understand the ethical and professional values that are expected of the profession	7) I believe I am adequately prepared to care for patients from different backgrounds	8) Generally I’m satisfied with the quality of my medical education
% of agree	TC	42.8	51.0	56.6	37.2	44.1	50.4	37.9	44.2
	RC	63.6	87.9	84.9	48.5	60.6	72.7	57.6	81.8
TC	M	3.23	3.38	3.54	3.21	3.30	3.43	3.20	3.28
	SD	0.965	0.898	0.841	0.904	0.930	0.864	0.955	0.886
RC	M	3.61	4.00	4.00	3.33	3.61	3.79	3.61	4.00
	SD	0.899	0.612	0.559	0.816	0.609	0.650	0.747	0.612
U (*p*)		1,874.5 (0.041)	*1,442.0 (<.0001)	*1,669.0 (0.003)	2,188.5 (0.417)	1,954.0 (0.078)	1,828.5 (0.023)	1,811.5 (0.021)	*1,308.0 (<.0001)
Cohen’s *d*		−0.407	−0.807	−0.644	−0.139	−0.394	−0.471	−0.478	−0.946

*stands for significant difference

*TC* traditional curriculum, *RC* reform curriculum

## Discussion

This graduation survey helped us answer questions about students’ perceptions of the reform and traditional curricula, whether the intended new elements were present in the new curriculum according to reform curriculum students, whether and how the two groups of students’ evaluations of their respective educational experience differed in several main curricular components, and what areas of the reform curriculum still needed improvement.

Traditional curriculum students’ satisfaction rates (between 37.9 % and 57.9 %) with their basic science learning were considerably lower than those of their reform curriculum peers (ranging from 69.7 % to 81.8 %). This result suggests that profound changes to the traditional curriculum were needed, and that reform has led to greater student satisfaction with the basic science part of their education. The significant differences between the views of the traditional and reform curriculum students also yields evidence that the new curricular goals of clarity in learning objectives, interdisciplinary integration, alignment of assessments to learning objectives, and relevance to clinical medicine were implemented somewhat effectively and present in the reform curriculum. The relatively low satisfaction with clinical relevance and preparation for clerkships indicates areas for further improvement.

The ratings of both traditional and reform curriculum students of their clinical science subjects were rather high. Compared with their traditional curriculum peers, reform curriculum students seemed more satisfied with their clinical science learning. Although the differences were not statistically significant, some may be practically significant given their effect sizes. This might be due to the fact that reform curriculum students were offered the year-long clinical science course *Clinical Pathophysiology and Therapeutics*, in which many clinical subjects were integrated and where clinical application was highly emphasized, and which was considered an outstanding course by reform curriculum students. Areas that need further innovations include rehabilitation, pharmacology, and evidence-based medicine, each with a satisfaction rate of 75 % or lower.

The multi-dimensional evaluation of six clerkships indicated that they were only somewhat satisfactory to students. Two clerkships that need the most improvement are community medicine and psychiatry clerkships, both of which are still among the newest and least mature of all clerkships. For all six clerkships, clarity of objectives and assessments of students’ performance are apparently weak areas.

Students’ evaluation of general medical education suggests that the reformed and traditional curriculum share some strengths and weakness with respect to the amount of training in clinical abilities. For example, patient interviewing skills and examination skills are considered ‘appropriate’ by the majority of students from both curricula, while care of ambulatory patients and health education are much less so. The finding that the views of students in the reform and traditional curriculum differed significantly regarding clinical reasoning was expected, as the reform curriculum placed more emphasis than the traditional curriculum on this area by means of clinical case or vignette discussions in all basic science and clinical science courses. Basic sciences in the reform curriculum were more clinically relevant (as evidenced in Table 1) and reform curriculum clinical science courses devoted more attention to clinical reasoning (as exemplified by the *Clinical Pathophysiology and Therapeutics* course). These reasons might also explain why the views of the two groups of students on diagnosis, though not statistically significant, have a relatively substantive effect size.

Regarding population health, large proportions of students deemed these areas ‘inadequate’. The significant difference between the views of reform and traditional curriculum on community medicine was as we had expected, as community medicine clerkship and learning activities were mandatory for reform curriculum students but optional for traditional curriculum students. The remaining areas did not receive added attention in the reform curriculum in spite of the reform. Further innovation efforts can prioritize some of these areas.

Finally, students’ assessment of their preparedness for residency and their rating of the overall quality of their medical school education perhaps present evidence that the new curriculum with its new teaching and learning methods may have led to students’ greater confidence in their abilities and greater satisfaction with their overall educational experience.

## Limitations of this study

Because the survey was voluntary and because the graduating students had met all programme requirements and were getting ready to leave the campus, many students did not respond to the invitation to participate. On the other hand, given the circumstances under which the survey was administered, the participation rate was quite high, perhaps because this was the first graduation survey ever conducted at the medical school and the students were eager to take the opportunity to voice their opinions.

We expected positive effects of the reform as it had caused profound curricular changes in content and structure based on up-to-date principles. However, other factors might have also contributed to the positive attitude among participants. For example, the reform curriculum was given additional attention and resources such as faculty training. The Hawthorne effect might have been a confounder, too – it was possible that the positive attitude of students following the reform curriculum resulted from the mere fact of being participants in the reform. Furthermore, there might be differences between the characteristics of the two groups of students, as it was possible that students in the reform curriculum were more enthusiastic about reform and therefore rated their reform curriculum relatively highly. Further investigations are necessary to fully determine what led to the greater satisfaction of reform curriculum students. Finally, satisfaction data are limited; without the support of other critical data sources such as learning outcomes, satisfaction data alone are not a sufficient basis for making educational change.

## Conclusion

Our general conclusion from this study is that although traditional curriculum students are somewhat satisfied with certain aspects of their curriculum, a thorough reform is called for by the traditional curriculum students. In addition, the response of reform curriculum students to the survey indicates that the reform has been implemented at least somewhat effectively, for the intended new elements are found to be present in the reform curriculum and reform curriculum students’ satisfaction is significantly higher in many areas of their education than that of their peers following the traditional curriculum.

Medical schools in many developing countries still rely heavily on traditional curricula and pedagogies and are in urgent need of change [15]. We hope that this study may offer encouragement and useful lessons to reformers in China and elsewhere, because it shows that comprehensive changes based on the state-of-the-art curricular and teaching principles can have the potential of improving student satisfaction and the quality of medical education in a cross-cultural context. The first key lesson from Wuhan University’s experience is that needs assessment is the important initial step in preparing for the reform, as it helps ‘diagnose’ the existing curriculum. Experts’ review, and student and faculty satisfaction surveys are two examples of assessment tools, both used by Wuhan University. When the reform is in progress, innovations should also be monitored by ongoing assessments to provide feedback for decision-makers and other stakeholders.

Second, a leadership committee must be in place to direct and implement the reform, and protected time for reform activities must be provided for key players, especially course directors.

Third, a borrowed foreign curriculum should be modified according to local contexts and needs. For case discussions, for example, Wuhan University teachers replaced some of the clinical cases used by the University of Chicago with ones collected from their teaching hospitals to make the cases locally relevant. As another example, topics that were not covered in the University of Chicago’s curriculum but would appear on China’s licensing exams were taught in Wuhan University’s new curriculum.

Fourth, provide training for faculty members to enhance their pedagogical knowledge and leadership skills. Training should respond to what faculty members need in order to effectively carry out the reform, and it can be done by regular workshops in the school and by faculty exchange between the collaborating institutions.

Finally, work toward sustaining the change. Successful change often takes longer than expected [16]. School leadership and faculty need to maintain their commitment to the reform to make the reform lasting. At Wuhan University, the work launched in 2009 has continued and has been enhanced ever since in spite of leadership successions. For more lessons from the Wuhan University experience, please see an article by Dong and colleagues [10].
